# In Patients Presenting with an ST-Elevation Myocardial Infarction, Overweight and Obese Patients Have the Lowest Inpatient Mortality whereas Cachexia Patients Have the Highest Inpatient Mortality Followed by Patients with Morbid Obesity

**DOI:** 10.3390/jcm13195897

**Published:** 2024-10-02

**Authors:** Mohammad Reza Movahed, Amir Parsa Abhari, Mehrtash Hashemzadeh

**Affiliations:** 1 Department of Medicine, College of Medicine, University of Arizona, Phoenix, AZ 85004, USA; 2 Department of Medicine, Sarver Heart Center, University of Arizona, Tucson, AZ 85724, USA

**Keywords:** obesity paradox, obesity, morbid obesity, weight categories, myocardial infarction, acute coronary syndrome, outcome

## Abstract

**Introduction:** The obesity paradox has been observed in patients with cardiovascular disease. The goal of this study was to evaluate whether obesity has a protective effect in patients presenting with an ST elevation myocardial infarction (STEMI). **Method:** Using the large Nationwide Inpatient (NIS) sample database, we evaluated the mortality in patients with a STEMI based on weight categories. **Results:** A total of 2,161,640 STEMI patients were found in the database over age 18. We found that overweight and obesity had the lowest mortality using univariate (overweight mortality of 5% vs. obesity mortality of 6.5% vs. 10.9% for normal weights) and multivariate analyses (overweight OR: 0.52, CI: 0.43–063; *p* < 0.001 and obesity OR: 0.7, CI: 0.67–0.74; *p* < 0.001), whereas cachexia was associated with the highest mortality in the univariate (cachexia 24.5%) and multivariate (OR: 2.28, CI: 2.13–2.44; *p* < 0.001) analyses, followed by morbid obesity in the multivariate analysis (OR: 1.07, CI: 1.02–1.12; *p* = 0.004). **Conclusions:** We observed a partial obesity paradox in patients with a STEMI showing that overweight have the best survival rates followed by obesity. Cachexia followed by morbid obesity had the lowest survival rates.

## 1. Introduction

The global burden of cardiovascular diseases (CVDs) continues to be a major cause of morbidity and mortality, with acute coronary syndrome (ACS) being one of the most critical presentations. One of the various types of ACS, an ST elevation myocardial infarction (STEMI), is characterized by an extended period of ischemia culminating in significant myocardial damage, which poses substantial risks for adverse outcomes and mortality [1]. The body weight and body mass index (BMI) are well-established modifiable risk factors for a variety of CVDs. The association between these anthropometric measures and cardiovascular outcomes has been revealed to a large extent, showing a complex or even paradoxical association, often referred to as the obesity paradox. This term indicates a better survival rate in overweight and obese subjects compared to their normal weight counterparts, particularly in certain individuals with established CVDs [2,3,4,5].

While the obesity paradox has been investigated in numerous contexts, its indication in the setting of a STEMI is still unexplained, and further research is warranted. Given the modifiable nature of the BMI and the reciprocal association of anthropometric indices with numerous factors including comorbid conditions, metabolic status, and the type of treatment, elucidating the true association of the BMI and mortality following a STEMI is of high importance. As such, in this study, we sought to explore the association between the BMI and mortality in STEMI patients by leveraging large-scale clinical data, as we are confident that our results could offer insights into adopting proper preventive and therapeutic strategies for the patients at a high risk of a STEMI or poor prognosis following a STEMI.

## 2. Materials and Methods

### 2.1. Setting and Data Collection

We performed a retrospective cohort study using the National Inpatient Sample (NIS), which comprises hospital inpatient databases from the Healthcare Cost and Utilization Project (HCUP). The NIS is intended to track national healthcare utilization trends, patient outcomes, and healthcare quality. It maintains a database of patients’ demographics and the reasons behind hospitalization through ICD-10 billing codes. By identifying specific ICD-10 codes, we extracted the relevant data from the corresponding billing codes for our study. The NIS database is available at www.hcup-us.ahrq.gov (accessed on 11 August 2024). This study contains the data from subjects aged over 18 years admitted to NIS hospitals from 2016 to 2020 and assigned ICD-10 diagnosis codes for a STEMI (I21.01, I21.02, I21.09, I21.11, I21.19, I21.21, I21.29, I21.3, I21.9, I21.A1, I21.A9, I22.0, I22.1, I22.5, and I22.9).

These patients were categorized based on their weights into four groups: cachexia (R64), overweight (E66.3), obesity (E66.9, E66.8, and E66.0), and morbid obesity (E66.01 and E66.2). The data regarding the comorbidities of the included patients were gathered as follows: diabetes, hypertension, chronic kidney disease (CKD), chronic obstructive pulmonary disease (COPD), an old MI, and cardiogenic shock. Moreover, we collected the data on demographic features, including age, sex, race, and length of stay, and detailed data respecting hospitalization, including insurance, hospital bed size, hospital location and region, and median household income. The outcome of this study was defined as death caused by cardiac dysfunction related to heart failure, ischemic heart disease, and arrhythmia; unwitnessed death was also considered to be the endpoint of our study.

### 2.2. Statistical Analysis

The demographic, clinical, and hospital characteristics are presented as the mean with standard deviation (SD) for the continuous variables and as proportions with 95% confidence intervals (CI) for the categorical variables. A logistic regression was performed to ascertain the odds of all-cause mortality over time. The analyses were implemented both in univariable and multivariable settings. All the statistical models were adjusted for confounding. All the analyses were conducted following the implementation of population discharge weights. All the *p*-values were 2-sided, and *p* < 0.05 was considered statistically significant. The statistical analyses were implemented using STATA 17 (Stata Corporation, College Station, TX, USA).

## 3. Results

A total of 2,161,640 STEMI patients over age 18 were found in the database. The detailed demographic and hospitalization data are thoroughly represented in Table 1, both for the total included population and subgroups classified according to the BMI. As shown in Table 2, Table 3, Table 4, Table 5 and Table 6, we found that the overweight and obesity subgroups had the lowest mortality using the univariate (odds ratio [OR] of overweight subgroup: 0.43 [CI: 0.35–0.52, *p* < 0.001] and OR of obesity subgroup: 0.57 [CI: 0.55–0.60, *p* < 0.001]) and multivariate analyses (OR of overweight subgroup: 0.52 [CI: 0.43–063, *p* < 0.001] and OR of obesity subgroup: 0.7 [CI: 0.67–0.74, *p* < 0.001]), whereas the cachexia subgroup was associated with the highest mortality in the univariate (OR: 2.65 [CI: 2.49–2.81, *p* < 0.001]) and multivariate (OR: 2.28 [CI: 2.13–2.44, *p* < 0.001]) analyses followed by morbid obesity in the multivariate analysis (OR: 1.07 [CI: 1.02–1.12, *p* = 0.004]). The cachexia subgroup had the highest mortality (24%) followed by the morbid obesity subgroup (9.3%), while the overweight population had the lowest mortality (5%).

## 4. Discussion

The findings of our study highlight the complex relationship between BMI and mortality in patients with a STEMI, contributing to the ongoing debate regarding the obesity paradox in cardiovascular conditions. Our analyses revealed that obese and overweight patients exhibited significantly lower mortality rates compared to their normal weight counterparts, while those with cachexia had the highest mortality rates. These results are in line with the notion of the obesity paradox, where a higher BMI appears to confer a survival advantage in certain CVDs. This term was initially used by Ellis et al. in 1996 reporting better clinical outcomes in obese and overweight patients with coronary heart disease undergoing a percutaneous coronary intervention (PCI) than those with a normal weight [6]. Since then, several studies have confirmed the so-called obesity paradox; however, some studies refuted this paradox and argued against the cardio-protective role of excess weight in patients with CVDs. In particular, Iliodromiti et al. found a 13% increase in CVD incidence per one SD increase in the BMI over 22 (5.2 for women and 4.3 for men) in a large database of 296,535 adults of white European descent without a baseline CVD [7]. Furthermore, Champagne-Langabeer et al. demonstrated significantly higher in-hospital mortality and door-to-balloon time in patients with a BMI > 40 and a STEMI who underwent a PCI through univariate linear and logistic regressions; these differences remained after multivariate adjustments for the door-to-balloon time, but not for mortality [8]. On the other hand, Baumann et al. demonstrated that with an increasing BMI, the rate of major adverse cardiac events decreased in 529 patients with a STEMI [9].

Regarding short-term mortality after a PCI, we conducted thorough research on the existing literature and we found homogenous findings indicating lower short-term mortality for the obese patients than the healthy weight ones [9,10,11]. These findings persisted in the comparison of overweight and healthy weight patients, except for in Wu et al., who conducted these analyses on an Asian population of 925 STEMI patients [12]. Reviewing the studies assessing the association of long-term mortality after a PCI and the BMI, Moscarella et al. revealed less frequent cardiac and all-cause deaths in obese patients than the overweight and healthy weight ones after five years of follow-up; however, when adjusting for confounding factors, the BMI was shown to be an independent predictor of all-cause death but not of cardiac death [13]. In line with these results, Joyce et al. displayed a worst overall survival in normal and underweight STEMI patients during a median follow-up of 5.2 years through a Kaplan–Meier analysis [14].

Consistently, Akin et al. demonstrated no significant difference in the rate of major adverse cardiac and cerebrovascular events, target vessel revascularization, and all-cause deaths between overweight and normal weight subjects undergoing a PCI after a 1-year follow-up [15]. These heterogeneous findings urged researchers to pool all the results of the relevant studies to deduce the true association between the BMI and STEMI prognosis, as, in this regard, Lie et al. yielded lower in-hospital, short-term, and long-term mortality in overweight and obese patients than healthy weight individuals with a STEMI undergoing a PCI [16]. Several theories could explain the obesity paradox following an AMI. Excess weight might provide nutritional reserves to endure acute stress and heightened metabolic demands, such as after an MI. In line with this theory, studies in intensive care units revealed that a higher BMI correlates with reduced hospital mortality and improved discharge outcomes in patients with various disorders, potentially justifying the protective effect of excess weight in frail MI patients [17,18]. Additionally, clinicians may treat post-MI obese patients more aggressively, respecting the obesity-related risks and morbidities.

Emerging evidence indicates that obesity may elicit inflammatory reactions and aggravate endothelial function, probably impacting the progression of atherosclerosis and the associated mortality risk [19]. Despite controversial findings, recent research has demonstrated that traditional inflammatory markers, namely C-reactive protein, are less effective predictors of atherosclerosis in obese individuals, who also tend to have better endothelial function compared to their non-obese counterparts [20,21]. These findings are further strengthened by additional recent studies, which suggest that obese patients with an acute MI present with less severe and complex coronary artery disease than non-obese ones, as assessed by quantitative coronary angiography metrics, such as SYNTAX scores and the number of stenotic lesions [22]. Strikingly, some studies have suggested that the concept of the obesity paradox might be better explained by the confounding effect of cachexia in normal weight patients rather than any certain advantage linked to obesity. Of note, our study supported the previous findings indicating a higher mortality and morbidity in cachectic patients with CVDs compared to normal weight ones. Cachexia is defined as a complex metabolic syndrome characterized particularly by muscle mass loss, which could accompany fat mass loss. The pivotal role of cachexia in the prognosis of patients with various conditions, including COPD, heart failure, and cancer, has been well studied [23]. The criteria used to define cachexia vary widely among the studies conducted in this regard. These definitions include a low BMI, recent weight loss, anorexia, fatigue and associated symptoms, low skeletal mass, and biochemical markers, or a combination of these factors, and the results of all these methods were relatively unified [24]. In other research conducted by the authors of this study, we have thoroughly explained whether the BMI is a proper option to be used in clinical practice and research to classify patients accordingly [25]. Our current knowledge is limited as to whether the concept of the obesity paradox relies on fat mass or lean mass, and the BMI does not appropriately distinguish between the variations of body composition and fat distribution. However, the BMI is still the first choice to be utilized in practice for large cohorts of patients [26].

Several explanations have been proposed to justify the poor prognosis of underweight patients with CVDs. One of these pertains to the augmented cardiometabolic demands following activated neurohormonal pathways and inflammatory reactions in patients with coronary artery disease [27]. Those with less subcutaneous fat and fewer energy reserves are less likely to properly counter the catabolic alterations subsequent to the infarction. Moreover, a vast majority of patients with a STEMI are previously diagnosed cases of chronic conditions, such as heart failure, CKD, DM, and COPD, and are prone to cardiac cachexia. The altered level of the endocrine mediators and biomarkers, including insulin, melanocortin, growth hormone, ghrelin, leptin, and neuropeptide Y, that are the known contributors to cardiac cachexia could explain the higher mortality in patients with this condition presenting with a STEMI [23,28]. Notably, experimental studies have demonstrated that cachexia and limited food consumption could induce structural and functional alterations even in normal hearts [29]. The other potential theory capable of justifying the high mortality in cachectic patients could be the likelihood of genetic predisposition among people with a low BMI to coronary artery disease. Since obesity is a well-known risk factor for a myocardial infarction, the higher mortality in underweight patients could shed light on the unidentified factors putting these patients at a higher risk of adverse outcomes after a STEMI. Furthermore, underweight patients are less likely to receive proper management following a STEMI, since these subjects suffer from a lack of functional reserves and are highly likely to present with lethal hemodynamic changes during an acute MI. They are also at a higher risk of developing procedure- or medication-related complications as, in this regard, studies have revealed higher rates of post-PCI bleeding and complications, potentially due to the smaller coronary vessels and the suboptimal artery-to-device ratio in underweight patients [30,31]. Interestingly, it has also been postulated that mortality and morbidity in the underweight population might be attributed to the limited efficacy or higher toxicity of the medications they receive [32].

While we have scrutinized and further elucidated the obesity paradox in a STEMI, this should not be misconstrued as a recommendation of behaviors that culminate in overweight or obesity in any population. Given that obesity is a major prominent risk factor for the development of various chronic conditions, including CVDs, those who adopt healthy habits are effectively following primary prevention against the onset of an MI. Taken together, we believe the results of our study and the evidence provided previously should be taken into consideration in light of precluding stringent weight-lowering strategies in patients with a STEMI or those who are susceptible to improve the short-term and long-term prognosis of the affected ones.

### Limitations

Several limitations account for this study, one of which is the retrospective nature of this study. We believe further prospective research is warranted to confirm our findings. The other limitation is the absence of the time to the event in our database, which impeded us from conducting survival analyses and further elucidating the role of the obesity paradox in the prognosis of STEMI patients. Moreover, we had no access to medications or the details of comorbidities in the analyses. Furthermore, we did not specify the mortality and the reason behind it, as we set all-cause mortality as the outcome of our study; we believe future research can resolve this limitation by repeating these analyses according to all-cause and cardiovascular mortality. However, we tried to overcome these drawbacks by including a large heterogeneous population in our study to strengthen our findings. The NIS database does not have data about physical fitness limiting our results.

## 5. Conclusions

Our study provides valuable insights into the intricate and multifaceted association between BMI and mortality in STEMI patients, conceding the obesity paradox. Overweight and obese individuals showed lower mortality, whereas cachexia was linked to the highest mortality rates, indicating the protective role of a higher BMI in a STEMI. However, this should not promote unhealthy weight gain, given the established detrimental effects of obesity in numerous conditions. Our findings suggest that strict weight-loss strategies may not be beneficial for all STEMI subjects, and a subtle approach to weight management is essential. Future studies should explore the potential underlying mechanisms of this paradox and consider individual patient profiles in treatment planning. Physicians should balance the benefits of preventing obesity-associated diseases with the protective aspects of a higher BMI in acute cardiovascular events. Eventually, adopting healthy lifestyle habits remains the bottom line for the primary prevention of CVDs and improving overall patient outcomes.

## Figures and Tables

**Table 1:** Demographic features of the included patients with a STEMI (LOS = length of stay, SD = standard deviation, IQR = interquartile range, and HMO = health maintenance organization).

2016–2020, Age above 18	Total STEMI	Normal Weight	Cachexia	Overweight	Obesity	Morbid Obesity
Total Population	2,161,640	1,757,675	29,980	11,700	215,210	148,930
Age (Median (IQR))	68(58–79)	69(59–80)	76(66–85)	64(55–74)	64(54–73)	62(53–71)
LOS (Median (IQR))	4(2–7)	4(2–7)	6(4–12)	3(2–6)	3(2–6)	5(2–9)
Total Charges USD (Median)	69610.5	68894	70427	73355	73490	72076
Mortality	10.56%	10.95%	24.56%	5.00%	6.58%	9.31%
Gender						
Male	60.39%	82.35%	1.22%	0.60%	10.05%	5.85%
Female	39.61%	79.73%	1.65%	0.45%	9.80%	8.48%
Race						
White	72.01%	81.42%	1.30%	0.54%	10.03%	6.79%
Black	13.03%	78.43%	1.94%	0.50%	10.27%	8.97%
Hispanic	8.46%	81.03%	1.13%	0.65%	10.70%	6.56%
Asian/PoC	2.76%	88.89%	2.18%	0.59%	5.68%	2.69%
Native American	0.68%	80.92%	1.16%	0.32%	9.98%	7.69%

**Table 2:** Univariate analysis results for each of the subpopulations according to BMI.

2016–2020	Mortality	Normal Weight Mortality	*p*-Value	Odds Ratio (CI)
Cachexia	24.56%	10.95%	<0.001	2.65 (2.49–2.81)
Overweight	5.00%	10.95%	<0.001	0.43 (0.35–0.52)
Obesity	6.58%	10.95%	<0.001	0.57 (0.55–0.60)
Morbid Obesity	9.31%	10.95%	<0.001	0.83 (0.80–0.87)

**Table 3:** All-cause mortality rate of cachectic patients along with the odds ratio according to the year.

Year	Mortality	Normal Weight Mortality	*p*-Value	Odds Ratio (CI)
2016	35.56%	11.93%	<0.001	4.07(3.17–5.24)
2017	27.09%	11.65%	<0.001	2.82(2.29–3.47)
2018	22.57%	10.16%	<0.001	2.58(2.28–2.92)
2019	23.14%	9.73%	<0.001	2.79(2.50–3.12)
2020	25.09%	12.04%	<0.001	2.45(2.21–2.71)

**Table 4:** All-cause mortality rate of overweight patients along with the odds ratio according to the year.

Year	Mortality	Normal Weight Mortality	*p*-Value	Odds Ratio (CI)
2016	6.28%	11.93%	0.008	0.49(0.29–0.83)
2017	4.75%	11.65%	<0.001	0.38(0.22–0.64)
2018	4.24%	10.16%	<0.001	0.39(0.26–0.58)
2019	2.88%	9.73%	<0.001	0.28(0.17–0.44)
2020	7.13%	12.04%	<0.001	0.56(0.41–0.76)

**Table 5:** All-cause mortality rate of obesity patients along with the odds ratio according to the year.

Year	Mortality	Normal Weight Mortality	*p*-Value	Odds Ratio (CI)
2016	6.81%	11.93%	<0.001	0.54(0.48–0.61)
2017	6.87%	11.65%	<0.001	0.56(0.50–0.62)
2018	5.96%	10.16%	<0.001	0.56(0.51–0.61)
2019	5.70%	9.73%	<0.001	0.56(0.52–0.61)
2020	7.54%	12.04%	<0.001	0.60(0.55–0.64)

**Table 6:** All-cause mortality rate of morbid obesity patients along with the odds ratio according to the year.

Year	Mortality	Normal Weight Mortality	*p*-Value	Odds Ratio (CI)
2016	11.86%	11.93%	0.92	0.99(0.86–1.14)
2017	10.40%	11.65%	0.05	0.88(0.78–1.00)
2018	8.30%	10.16%	<0.001	0.80(0.73–0.88)
2019	7.86%	9.73%	<0.001	0.79(0.72–0.86)
2020	10.30%	12.04%	<0.001	0.84(0.78–0.90)

## Data Availability

NIS data base is publicly available upon purchase.
